# Construction and Immunogenicity Evaluation of a Recombinant Infectious Bronchitis Virus H120-Based Vaccine in Broiler Chickens

**DOI:** 10.3390/ani16020336

**Published:** 2026-01-22

**Authors:** Ali Nayef, Sara Jibreen, Mustafa Ababneh

**Affiliations:** 1 Department of Research and Development, The Arab Pesticides and Veterinary Drugs Manufacturing Company, El-Hassan Industrial Estate, Ar-Ramtha 11193, Jordan; aali4163@gmail.com (A.N.); vetsarajibreen00@gmail.com (S.J.); 2 Department of Basic Medical Veterinary Sciences, Faculty of Veterinary Medicine, Jordan University of Science and Technology, Irbid 22110, Jordan

**Keywords:** Infectious Bronchitis Virus, recombinant vaccine, reverse genetics, Golden Gate Assembly, immunogenicity

## Abstract

Infectious Bronchitis Virus is one of several major viral infections in poultry, affecting the respiratory, reproductive, and renal systems and causing significant economic losses worldwide. Current vaccines, including the H120 strain, provide limited cross-protection against emerging variants, underscoring the need for improved vaccine strategies. In this study, the complete genome of IBV H120 was divided into 12 fragments, synthesized, and assembled using the Golden Gate Assembly (GGA) method. The recombinant virus (rH120) was successfully rescued in chicken fibroblast cells and propagated in embryonated specific-pathogen-free (SPF) chicken eggs. Growth kinetics in embryonated SPF chicken eggs revealed similar replication patterns between rH120 and the original H120 strain. In broiler chickens, rH120 replicated efficiently, as confirmed by viral RNA detection in throat and cloacal swabs, and induced a stronger antibody response by 14 days post-infection. The rH120 virus proved to be genetically stable, infectious, and immunogenic, indicating that GGA-based reverse genetics is an effective system for IBV vaccine development.

## Simple Summary

Infectious Bronchitis Virus (IBV) remains a major threat to poultry production due to its genetic diversity and the limited cross-protection of current vaccines. In this study, we developed a recombinant H120 virus (rH120) using a Golden Gate Assembly-based reverse genetics system that assembled 12 synthetic genome fragments. The virus was successfully rescued in chicken fibroblast cells, propagated in embryonated SPF eggs, and showed growth characteristics similar to the original H120 strain. In broiler chickens, rH120 replicated efficiently and induced a strong antibody response, confirming its immunogenicity. These results demonstrate that Golden Gate Assembly provides a robust platform for generating genetically defined IBV vaccine candidates.

## 1. Introduction

Infectious Bronchitis Virus (IBV) is a highly contagious avian coronavirus that affects the respiratory, reproductive, and renal systems of chickens, causing major economic losses to the poultry industry worldwide [1]. The disease can impact birds of all ages and production types, leading to poor growth, reduced egg production and quality, and greater vulnerability to secondary bacterial infections [2]. IBV belongs to the genus *Gammacoronavirus* within the family *Coronaviridae*. Its genome consists of a positive-sense, single-stranded RNA that encodes four major structural proteins: the spike (S), envelope (E), membrane (M), and nucleocapsid (N) proteins. The spike glycoprotein (especially its S1 subunit) is a key target for neutralizing antibodies and plays a central role in viral attachment and tissue tropism [3].

Due to its high genetic and antigenic diversity, IBV remains endemic and a major threat to the poultry industry worldwide even with extensive vaccination regimes [4]. The intrinsic and frequent virus mutations, especially in the spike protein gene along with the recombination events between different IBV strains, gives rise to various genotypes and serotypes that might provide limited cross-protection [5,6,7]. The Massachusetts serotype strain H120 is one of the most widely used live-attenuated vaccines and serves as the foundation of many vaccination schedules [8,9,10]. However, it provides variable degrees of protection against heterologous and newly emerging variants, such as QX (G1-19) and variant-2 (GI-23) strains, which are becoming increasingly prevalent in the Middle East and Asia [11,12,13,14]. This ongoing emergence of diverse field strains underscores the need for more adaptable vaccine platforms that can be rapidly updated and precisely engineered.

Recent advances in reverse genetics have transformed the study of RNA viruses, enabling complete genome reconstruction and the creation of recombinant viruses with defined genetic compositions [15,16]. Among these techniques, Golden Gate Assembly (GGA) offers a fast, cost-effective, and highly accurate approach for assembling full-length viral genomes from multiple synthetic fragments using type IIS restriction enzymes [17,18]. This method has proven valuable for studying viral gene function, understanding pathogenesis, and designing improved vaccines [19,20]. While reverse genetics systems have been developed for several IBV strains [21,22,23], to date, no studies have used Golden Gate Assembly to reconstruct the classical H120 vaccine strain or to evaluate its biological and immunological properties in chickens.

In this study, we constructed a recombinant H120 strain of IBV (rH120) using a Golden Gate Assembly–based reverse genetics system. We examined its replication and growth characteristics in embryonated SPF chicken eggs and evaluated its immunogenicity in broiler chickens. The successful rescue and evaluation of rH120 demonstrate the reliability of Golden Gate Assembly for generating genetically defined IBV viruses and highlight its potential as a platform for developing next-generation, genotype-matched vaccines to better control infectious bronchitis in poultry.

## 2. Materials and Methods

The Scientific Research Ethics Committee of Ahliyya Amman University reviewed and approved all procedures carried out in the current study (AUP: AAU/03/02/2025-2026).

### 2.1. Virus, Cells, and Eggs

The IBV H120 strain was used as the parental genetic backbone. The complete genome of this virus was obtained from GenBank (accession number FJ807652). Twelve genomic fragments covering the entire viral genome were synthesized and assembled using Golden Gate Assembly, and they were rescued using chicken embryo fibroblast cells (CEFs) and embryonated SPF chicken eggs at 9 days of age (VALO BioMedia GmbH, Osterholz-Scharmbeck, Germany). A commercial H120 vaccine was used in both growth kinetic and chicken infection experiments. Primary chicken embryo fibroblasts obtained from 9-day-old chicken embryos and BHK-21 cells were maintained in Dulbecco’s Modified Eagle Medium (DMEM) (Thermo Fisher Scientific, Waltham, MA, USA) supplemented with 10% fetal bovine serum (Cytiva, Vienna, Austria), 100 units mL^−1^ penicillin, and 100 µg mL^−1^ streptomycin (Euroclone, Pero, Italy) and kept at 37 °C in a CO_2_ incubator.

### 2.2. Design of Recombinant H120 by Golden Gate Assembly Strategy-Based Reverse Genetics

Using NEBridge SplitSet^®^ Tools available at the New England Biolabs website and the Golden Gate Assembly tool in the SnapGene^®^ software version 8.2.1 (GSL Biotech LLC, Chicago, IL, USA), the entire H120 genome was split into 12 fragments of various sizes. Fragment 10 was designed to have the full length of the coding sequence of the *spike* gene. Special features were included in the design of those 12 fragments to ensure efficient transcription of the rH120 genome. Fragment 1 contains the T7 promoter sequence. In fragment 12, a 30-nucleotide poly (A) tail was added directly after the 3′ untranslated region (UTR). This was followed by the insertion of a Hepatitis Delta Virus (HDV) ribozyme sequence and then a T7 transcription terminator positioned downstream of the ribozyme. To complete the design, fusion sites were added to the 5′ and 3′ ends of the construct, allowing it to form a circular assembly. GGA employs Type IIS restriction enzymes, such as *BsmBI* (used in our strategy), which cleave outside their recognition sequences, enabling multiple DNA fragments to be assembled efficiently in a single reaction. Because the H120 genome contains four internal *BsmBI* recognition sites, these sites were removed by introducing silent point mutations at nucleotide positions 5159 (C→T), 7363 (G→C), 15,461 (C→G), and 26,079 (C→G). These substitutions did not alter the amino acid sequence and were designed according to the chicken codon usage.

All 12 fragments of the IBV H120 strain were chemically synthesized and cloned into pUC57-mini plasmids by GenScript (Piscataway, NJ, USA). The plasmids were produced at an industrial scale and supplied as high-purity, endotoxin-free DNA preparations.

### 2.3. Golden Gate Assembly of IBV H120 Strain, Transfection, and Virus Rescue

All 12 plasmids of the 12 fragments were used in the GGA reaction. The GGA reaction was performed in a final volume of 20 μL, containing 3 nM of each DNA fragment (measured by Qubit 4), and 2 μL of the NEBridge Golden Gate Assembly Kit (BsmBI-v2) (NEB #E1602) (New England Biolabs, Ipswich, MA, USA) in 1× T4 DNA Ligase Reaction Buffer. The reaction mixtures were subjected to 90 thermal cycles, alternating between 37 °C and 16 °C for 5 min each and followed by a final incubation at 60 °C for 5 min. The assembled products were then stored at −20 °C until their use in the virus recovery experiments. A total of 90 cycles was applied to maximize the yield of the full-length assembled construct. For transfection and virus rescue, CEFs were seeded in six-well plates and cultured in Dulbecco’s Modified Eagle Medium (DMEM; Thermo Fisher Scientific, Waltham, MA, USA) supplemented with 10% fetal bovine serum (Cytiva, Vienna, Austria), 100 U mL^−1^ penicillin, and 100 µg mL^−1^ streptomycin (Euroclone, Pero, Italy). Cells were maintained at 37 °C in a humidified atmosphere containing 5% CO_2_ until they reached 70–80% confluence. The monolayers were then infected with vaccinia virus at a dose of 10^3.7^ TCID_50_ per well and incubated at 37 °C with 5% CO_2_ for 1.5 h to allow viral adsorption.

After incubation, the inoculum was removed and replaced with Opti-MEM medium (Thermo Fisher Scientific, Waltham, MA, USA) containing 12 µL of Lipofectamine 2000 (Thermo Fisher Scientific, Waltham, MA, USA), 20 µL of the GGA reaction mixture, and 2.5 µL of the helper plasmid carrying the *nucleocapsid* gene.

For transfection, two solutions were prepared separately:

Solution A: 20 µL of the GGA reaction mixture and 2.5 µL of the *nucleocapsid* gene helper plasmid was diluted in 1.5 mL of Opti-MEM.
Solution B: 12 µL of Lipofectamine 2000 was diluted in 1.5 mL of Opti-MEM.

Both solutions were incubated at room temperature for 15 min, combined, and then left for an additional 30 min to allow complex formation. Meanwhile, vaccinia virus–infected cells were washed twice with Opti-MEM to remove residual inoculum.

Next, 3 mL of the transfection mixture was added to each well and incubated at 37 °C with 5% CO_2_ for 90 min. The mixture was then replaced with DMEM and the cells further incubated for 48 h under the same conditions. Following incubation, the cells underwent three freeze–thaw cycles (−80/37 °C) and the harvested supernatant was stored at −20 °C.

A 500 µL aliquot of the harvested material was subsequently inoculated into 9-day-old embryonated SPF chicken eggs for three blind passages. Finally, the allantoic fluid was collected and stored at −20 °C for further analyses.

### 2.4. Validation of GGA Reaction

Four PCR reactions were performed to assess the success of the GGA reaction. The first and second PCR protocols amplify 518 bp and 151 bp products, spanning the region fragments 1, 2 and 3, 5, and 6, respectively. The third PCR reaction was obtained from [24] to detect the production of gene 5 and gene 7 from the subgenomic RNA of rH120 virus (producing two products at 500 bp and 2000 bp, respectively). The fourth PCR reaction was designed to amplify a 366 bp region in the *nucleocapsid* gene that has the silent mutation of C to G at position 26,079.

#### 2.4.1. Viral RNA Extraction and Complementary DNA Synthesis

Viral RNA was extracted using Quick-RNA Viral Kit (Zymo Research Co., Irvine, CA, USA) following the manufacturer’s recommendations. 200 µL of the allantoic fluid were mixed with 200 µL of DNA/RNA shield; then, 800 µL of viral RNA buffer was added and the mixture transferred into the zymo-spin IC Column. Following centrifugation for 2 min, 500 µL of viral wash buffer was followed by centrifugation for 1 min. This step was repeated twice before washing in 500 µL ethanol (100%) and 1 min centrifugation. RNA was eluted by adding 15 µL of DNase/RNase-free water directly in the column matrix, which was centrifuged again for 30 s and stored at −80 °C. The cDNA was then synthesized using the All-in-One 5x RT Master Mix kit (abmgood, Richmond, BC, Canada) using the following steps. 4 µL of All-in-One 5x RT Master Mix was mixed with 2 µg of the extracted RNA and 14 µL of nuclease-free water (NFW). The reaction was carried out at 37 °C for 15 min followed by 60 °C for 10 min. The mixture was stored at −20 °C.

#### 2.4.2. Conventional PCR Protocols

The primer sequences for the first and second PCR protocols, which span fragments 1, 2 and 3, 5, and 6, respectively, are: 1F: 5′-TGCACCATCTTGAGTTGCCTA-3′, 3R: 5′-ATTGCGGCATAAAGCCAT-3-, 5F: 5′-ACTGCTTGTTTTGCCGTGTC-3′, and 6R: 5′-CCCAGTCTGCAGATTACGGT-3′-. 10 pmol from each primer was mixed with 10 µL of Taq2x PCR Master Mix, and 2 µL of the cDNA sample, and diluted by nuclease-free water up to 20 µL. The PCR thermal conditions were 95 °C for 3 min followed by 40 cycles of 95 °C for 30 s, 60 °C for 30 s, 72 °C for 1 min, and a final cycle of 72 °C for 2 min. For the third PCR reaction, primer sequences were obtained from [24] and the PCR thermal conditions were as mentioned above. For the fourth PCR, the primer sequences are *nucleocapsid* gene sequence F: 5′-CTATTGCACTAGCCTTGCGC-3′ and *nucleocapsid* gene sequence R: 5′-TTCTCTAACTCTATACTAGCC-3′. 10 pmol from each primer was mixed with 10 µL Taq2x PCR Master Mix and 2 µL of the cDNA sample and diluted by nuclease-free water up to 20 µL. The PCR thermal conditions were 95 °C for 3 min followed by 40 cycles of 95 °C for 30 s, 62 °C for 30 s, 72 °C for 1 min, and a final cycle of 72 °C for 2 min. After completion of the PCR reactions, the products were run in a 1.5% agarose gel electrophoresis set-up and images taken. The products of the fourth PCR reaction were sent for Sanger sequencing at Macrogen (Seoul, Republic of Korea). Sequences were viewed and edited using the Finch TV software version 1.4.0 (Geospiza, Inc., Seattle, WA, USA).

#### 2.4.3. Recombinant H120 (rH120) Viral Propagation and Titration

To determine the virus titer, rH120 was inoculated in 9-day-old embryonated SPF chicken eggs via the allantoic cavity and incubated at 37 °C for 48 h. After chilling at 4 °C overnight, the infected allantoic fluid was harvested. Viral titers were then determined by inoculating 10-fold serial dilutions (10^−1^ to 10^−6^) of the virus into five 9-day-old embryonated SPF eggs per dilution via the allantoic cavity. Inoculated eggs were monitored for embryo mortality for 7 days at 37 °C and the 50% egg infectious dose (EID_50_/mL) was calculated using the Reed–Muench method.

### 2.5. Growth Kinetics of Recombinant H120 in Embryonated SPF Chicken Eggs

Both original H120 and rH20 viruses were inoculated at a titer of 10^3^ EID_50_ into the allantoic cavity of 9-day embryonated SPF chicken eggs. Allantoic fluid was harvested from five eggs inoculated with each virus at 12, 24, 36, and 48 h. Growth kinetics were assessed using RT-qPCR and expressed as log_10_ of RNA copies/reaction.

#### Reverse Transcriptase-Quantitative PCR (RT-qPCR)

Viral RNA was extracted from the allantoic fluid using the Quick-RNA Viral Kit (Zymo Research Co., Irvine, CA, USA) as mentioned above. RNA concentration was measured using a BioTek PowerWave XS2 spectrophotometer (BioTek Instruments, Inc., Winooski, VT, USA) and the concentrations of all samples were subsequently normalized at 100 ng/µL. Complementary DNA (cDNA) was synthesized using the GoScript™ Reverse Transcription System (Promega, Cat. No. A5003) (Promega, Madison, WI, USA). For each reaction, two separate mixtures were prepared as follows:

Solution A: Experimental RNA (100 ng per reaction) was mixed with 0.5 µL of Oligo(dT)_18_ primer and 0.5 µL of random primer. Nuclease-free water was added to a final volume of 5 µL. The mixture was incubated at 70 °C for 5 min, then immediately placed on ice for 5 min where it remained until use in the reverse transcription reaction.

Solution B: In a separate tube, nuclease-free water was added to achieve a total reaction volume of 15 µL along with 4 µL of GoScript™ 5× Reaction Buffer, 2 µL of MgCl_2_, 1 µL of PCR Nucleotide Mix (0.5 mM of each dNTP), and 1 µL of GoScript™ Reverse Transcriptase. All components were gently mixed in the order listed and kept on ice until combined with Solution A. Reverse transcription was performed under the following thermal conditions: 25 °C for 5 min, 42 °C for 60 min, and 70 °C for 15 min to inactivate the enzyme. The resulting cDNA was stored at −20 °C for further analyses.

For RT-qPCR, a standard curve was generated by serial dilution of a plasmid containing a fragment of the *nucleocapsid* gene cloned in pMG plasmid (Macrogen, Seoul, Republic of Korea). The primer sequences used in the qPCR were NqPCR F: 5′-CCTGGAAACGAACGGTAGAC-3′ and NqPCR R: 5′-CTGGCATCTTTATACCTACTCTAAACT-3′. 10 pmol from each primer was mixed with 10 µL of BlasTaq2x qPCR Master Mix and 2 µL of the cDNA sample and diluted by nuclease-free water up to 20 µL. The qPCR conditions were 95 °C for 3 min followed by 40 cycles of 95 °C for 15 s and 60 °C for 1 min. The qPCR was run in Rotor-Gene Q Thermal Cycler (Qiagen, Hilden, Germany). The results were expressed as log_10_ of RNA copies/reaction.

### 2.6. Infection of Broiler Chicken with Recombinant rH120 Virus

Thirty 21-day-old broiler chickens (Ross 308 breed) from a local supplier were allocated into three groups: 12 birds in Group 1; received the IBV rH120 vaccine, 12 birds in Group 2 received; the IBV H120 vaccine, and 6 birds in Group 3 received only PBS. In the shedding experiment, four chickens from each group were labelled and sampled from both the throat and cloaca at three time points (3, 6, and 10 dpi). For blood sampling (0, 7, and 14 dpi), eight chickens were labelled and sampled in each of the vaccinated groups, while six chickens were sampled in the PBS control group. the same birds were sampled longitudinally at 3, 6, and 10 dpi for viral shedding and at 0, 7, and 14 dpi for serology.

Broiler chickens at 21 days of age were housed in biosafety level 3 isolators. Two groups were inoculated by ocular route with 10^5^ EID_50_ of either rH120 or H120 virus. The third group was left as a control group and inoculated with PBS only. Indirect ID Screen^®^ Infectious Bronchitis ELISA kit was used to detect antibodies against IBV (Innovative Di-agnostics, Grabels, France). The ELISA procedure was done according to the manufacturer’s manual, and the results were expressed as optical densities (OD) at 450 nm. To evaluate the shedding of the IBV viruses (rH120 or H120), throat and cloacal swabs were collected in RNA shield solution. RT-qPCR was performed as mentioned in the growth kinetics section.

### 2.7. Statistical Analysis

The experimental unit was defined as the individual chicken for analyses of ELISA antibody responses and viral shedding, and as the individual embryonated SPF egg for viral growth kinetics experiments. Viral RNA copy numbers were log_10_-transformed prior to analysis to stabilize variance and approximate normality. Given the inherently limited sample sizes associated with controlled in vivo experiments, Welch’s correction was applied to account for potential heterogeneity of variances between groups. A formal a priori power analysis was not performed; however, sample sizes were selected based on established and widely accepted experimental designs commonly used in IBV vaccine evaluation studies. Comparisons between the two groups at each time point were made using an unpaired, two-tailed Student’s *t*-test with Welch’s correction to account for possible differences in variance between groups.

A *p*-value of less than 0.05 was considered statistically significant. Levels of significance are indicated in the figures as follows: *p* < 0.05, *p* < 0.01, *p* < 0.001, *p* < 0.0001 (****), and *p* ≥ 0.05 (ns, not significant).

## 3. Results

### 3.1. Construction and Rescue of Recombinant H120 (rH120) Virus

The full-length genome of the IBV H120 strain was successfully reconstructed using a GGA strategy with an assembly fidelity of 97% as calculated by the GGA function in SnapGene software (https://www.snapgene.com/). All 12 designed fragments, including a modified *spike* gene encoded entirely in fragment 10, were efficiently synthesized and assembled into a circular construct. Silent mutations were introduced to eliminate internal *BsmBI* restriction sites without altering the amino acid sequence.

Following transfection of vaccinia virus-infected CEF cells with the GGA product and helper plasmid encoding the *nucleocapsid* gene, cytopathic effects were observed after 48 h. The resulting lysates were passaged in 9-day-old embryonated SPF chicken eggs. After three blind passages, viable virus was successfully recovered in the allantoic fluid, confirming the rescue of the recombinant rH120 virus.

### 3.2. Validation of Viral Genome Assembly

**Figure 1:**
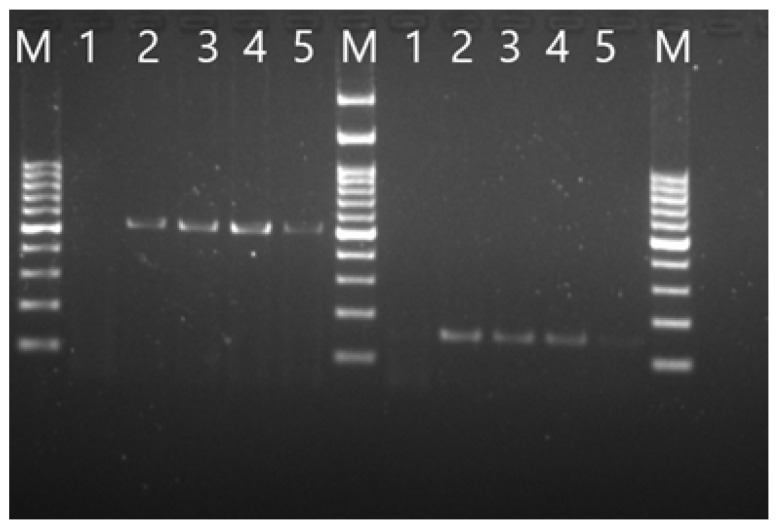
Agarose gel electrophoresis showing PCR amplification of recombinant IBV H120 genome fragments. Two PCR reactions were performed to amplify regions spanning fragments 1–3 and 5, 6, yielding the expected products of 518 bp and 151 bp, respectively. M: 100 bp DNA marker. For both PCR results: lane 1, negative control; lanes 2 and 3 are H120; lanes 4 and 5 are rH120.

**Figure 2:**
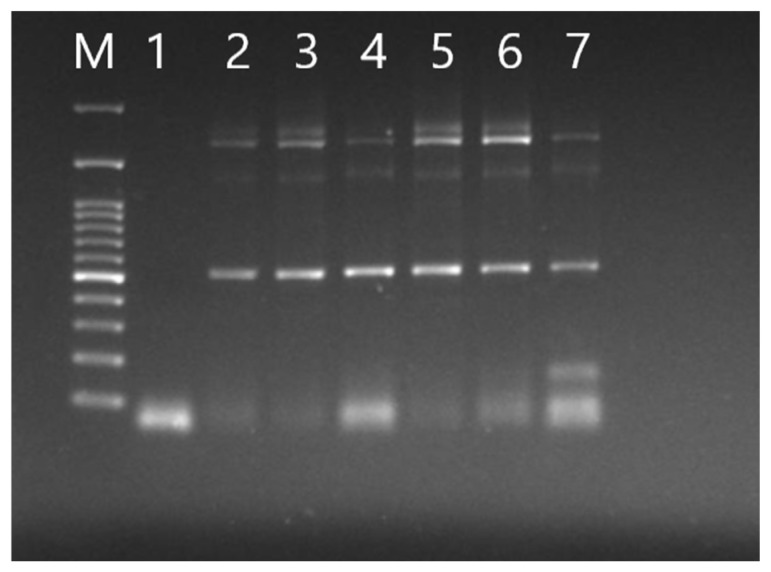
Agarose gel electrophoresis showing subgenomic PCR amplification of gene 5 and gene 7 from rH120-infected samples. The reactions produced the expected bands of approximately 500 bp and 2000 bp, respectively, confirming transcription of the corresponding subgenomic RNAs. M: 100 bp DNA marker; lane 1, negative control; lanes 2 and 3 are H120; lanes 4–7 is rH120.

**Figure 3:**
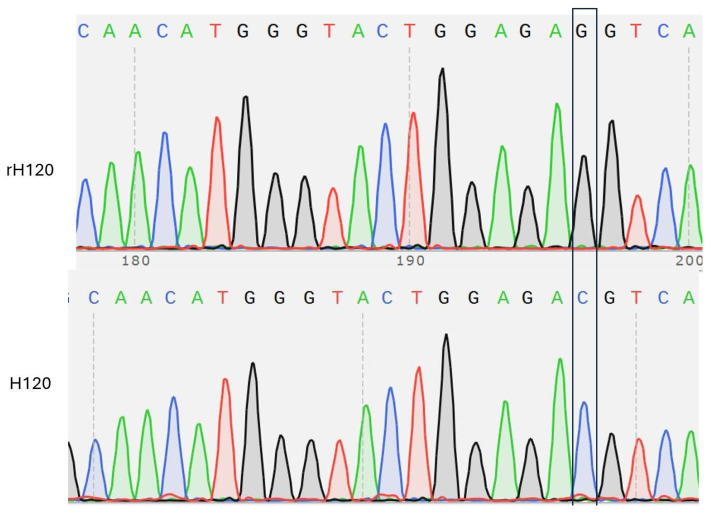
Comparative chromatogram analysis of the *nucleocapsid* gene region in rH120 and the original H120 strain. A single-nucleotide substitution (C→G) at position 26,079, highlighted in the boxed region, was identified in the rH120 sequence, confirming the successful introduction of this silent mutation to eliminate the internal *BsmBI* recognition site during the recombinant virus construction.

Multiple PCR reactions confirmed correct assembly and transcription of the recombinant virus. Two PCR reactions targeting regions spanning fragments 1, 2, 3, and 5, 6 produced expected amplicons of 518 bp and 151 bp, respectively, as shown in Figure 1. Subgenomic mRNA of gene 5 and gene 7 was also detected in infected samples, yielding 500 bp and 2000 bp products, respectively (Figure 2). A fourth PCR targeting the *nucleocapsid* gene confirmed the presence of the engineered C→G mutation at position 26,079. Sanger sequencing of the amplified region validated this mutation, confirming the identity of the recombinant virus (Figure 3).

### 3.3. Propagation and Titration of rH120 Virus

**Figure 4:**
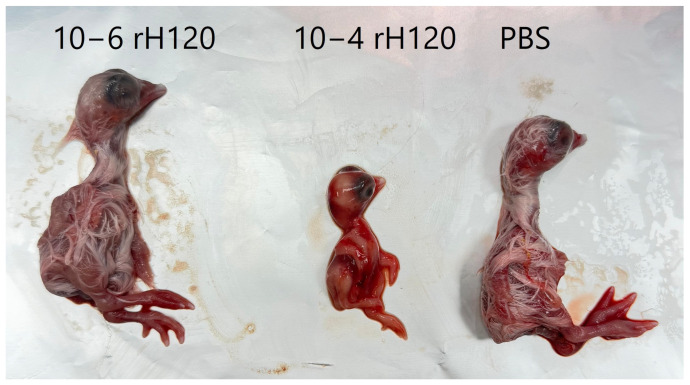
Titration of rH120 virus in 9-day-old embryonated SPF chicken eggs. Embryos inoculated with the 10^−4^ dilution of rH120 showed clear signs of dwarfing and hemorrhages while those inoculated with the higher dilution (10^−6^) appeared similar to the PBS-inoculated controls.

The rescued rH120 virus propagated efficiently in embryonated SPF chicken eggs. Following inoculation and incubation, allantoic fluid was collected, and viral titers were calculated using the Reed–Muench method (Figure 4). The mean titer across experiments reached approximately 10^5^ EID_50_/mL, demonstrating robust replication comparable to the commercial H120 strain.

### 3.4. Growth Kinetics in Embryonated SPF Chicken Eggs

**Figure 5:**
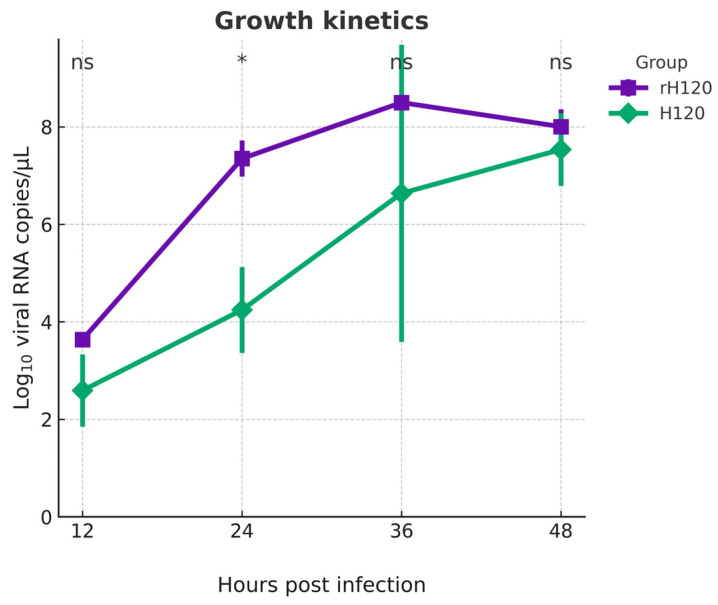
Growth kinetics of both the rH120 (purple line) and original H120 (green line). Both viruses were inoculated into 9-day-old embryonated SPF chicken eggs and showed similar replication kinetics, with rH120 having slightly higher viral RNA levels at 24 h post-inoculation. By 36 and 48 h, the differences between the two strains were statistically insignificant. Asterisks (*) indicate statistically significant differences (*p* < 0.05), ns (not significant).

To make sure that the generated recombinant virus has the ability to replicate efficiently in chicken eggs, both rH120 and H120 viruses were inoculated into 9-day-old embryonated SPF chicken eggs with 10^3^ EID_50_. Using RT-qPCR, the viral RNA amounts were expressed as log_10_ viral RNA copies/mL and determined at 12, 24, 36, and 48 h post-inoculation (Figure 5). rH120 exhibited a markedly higher viral RNA level at 24 hpi (*p* < 0.05), and a still detectable, but reduced difference at 36 hpi, before convergence at later time points. Both viruses reached their peaks level at 36 h after inoculation. The growth kinetics experiment clearly shows that rH120 and H120 have similar replication kinetics in embryonated SPF chicken eggs as in the original H120 virus.

### 3.5. In Vivo Infection and Immune Response in Broiler Chickens

**Figure 6:**
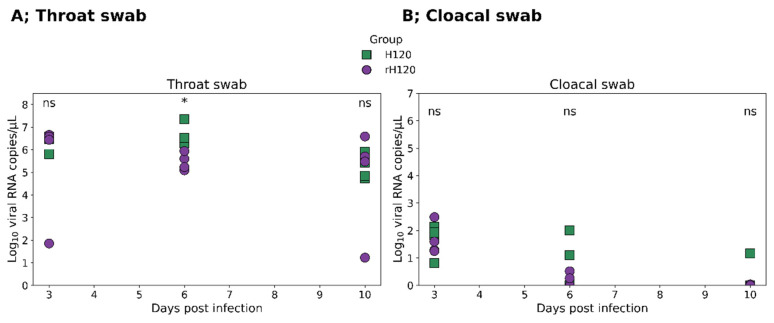
Viral RNA shedding profiles in throat and cloacal swabs of broiler chickens experimentally infected with recombinant rH120 and original H120 viruses. (**A**) Throat swabs: four samples were collected at each time point. Both groups showed detectable viral RNA beginning at 3 days post-infection (dpi). The viral load peaked at 6 dpi, where birds inoculated with the recombinant rH120 (purple circles) had significantly higher RNA copies compared to those infected with the original H120 strain (green squares). By 10 dpi, viral levels declined in both groups, with no significant differences. (**B**) Cloacal swabs: four samples were collected at each time point. Intermittent viral shedding was detected from 3 to 10 dpi in both groups, but the levels were generally lower than those observed in the throat samples, and no significant differences were found between rH120 and H120. (*): *p* < 0.05 (statistically significant), ns (not significant). At 3 dpi, viral IBV RNA was detected in all four sampled birds in both groups. However, at 6 and 10 dpi, only two of the four sampled birds in each group had detectable IBV RNA. Notably, at 10 dpi, among the two positive birds in each group, one bird exhibited a very low viral RNA level, whereas the other showed an unexpectedly high viral RNA level, representing an extreme outlier. These outlier data points were excluded from the analysis.

Three groups of broiler chickens at 21 days of age were inoculated with either rH120, H120, or PBS. This experiment was designed to check the in vivo viral replication (measured by viral shedding) and the ability of the rH120 and H120 to induce humoral immune response (as measured by IBV-specific antibody titer). To assess viral shedding, throat and cloacal swabs were collected at 3, 6, and 10 dpi and viral RNA measured by RT-qPCR (Figure 6). Both rH120 and H120 groups exhibited similar shedding profiles.

In throat swabs, four samples were collected at each time point. Viral RNA levels were comparable at 3 dpi, but significantly higher throat shedding was observed in the rH120 group at 6 dpi followed by a marked decline at 10 dpi, consistent with Figure 6A. In cloacal swabs, four samples were collected at each time point. At 3 dpi, all four swabs tested positive for viral RNA in both groups, whereas at 6 dpi and 10 dpi, only two of the four swabs yielded a detectable viral titer. Overall, viral shedding was consistently higher through the respiratory route than through the cloacal route. No viral RNA was detected in the negative control group. All viral RNA quantities are expressed as log_10_ viral RNA copies/mL as determined by RT-qPCR.

**Figure 7:**
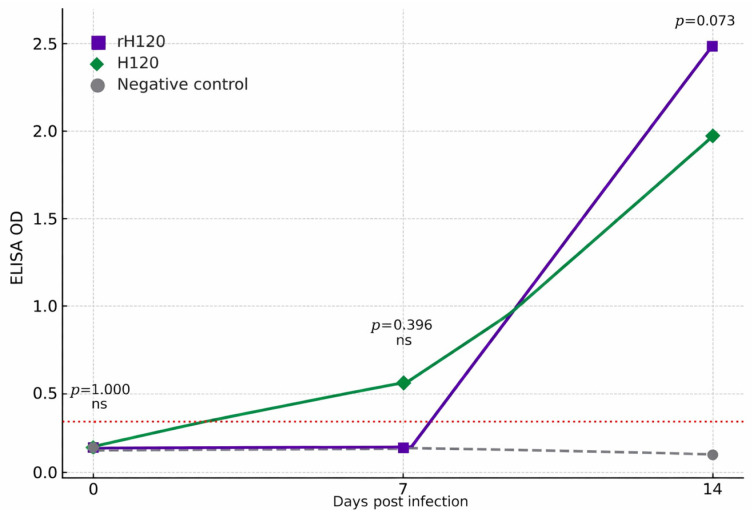
IBV-specific antibody response in broiler chickens infected with rH120 and H120 viruses as measured by ELISA. Data is expressed as OD at 450 nm. Serum samples were collected at three points (3, 7, and 14 dpi). The purple line expresses the increase in IBV-specific antibody titer for the rH120 while the green line expresses the original H120 virus. The negative group was represented by the grey line. The IBV-specific antibody titer increased by day 7 and at 14 dpi there was a marked increase in the antibody titer for both viral groups compared to the negative group. The red dotted line represents the assay’s positive cut-off value (OD = 0.25), ns (not significant).

ELISA testing revealed a strong serological response in both groups (rH120 and H120) with antibody levels significantly increasing at 7 and 14 days post-infection compared to the control group. No antibodies were detected in PBS-inoculated birds (Figure 7).

## 4. Discussion

The aims of this study were to (1) reconstruct the full-length genome of the Infectious Bronchitis Virus H120 strain using a GGA-based reverse genetics system, and (2) to rescue a viable, recombinant virus, designated as rH120. The 12 genomic fragments of H120 were assembled using GGA, including the *spike* gene in fragment 10. The silent mutations introduced to remove internal *BsmBI* restriction sites did not alter the encoded amino acids or virus viability. The infectious virus was recovered successfully following transfection; in addition, serial passage in embryonated SPF eggs confirmed that the reverse genetics system is functional and reliable for IBV manipulation.

In this study, the choice of reverse genetics system was based on its suitability for vaccine development and mutagenesis studies. Several platforms have been developed for IBV and other coronaviruses. The most commonly used approaches include Golden Gate Assembly [19], targeted RNA recombination [21], vaccinia virus vectors [22], bacterial artificial chromosome (BAC)-based systems [25], and transformation-associated recombination (TAR) cloning [23,26]. GGA was selected as it offers rapid, modular, and scar-free assembly.

Sequencing and molecular verification by PCR were used to confirm the genetic integrity of the rescued virus. The accuracy of genome transcription and viral replication was demonstrated by (1) the presence of expected amplicons, which span different genomic regions, and (2) the detection of subgenomic mRNAs. The single-nucleotide substitution (C→G) at position 26,079 was introduced to remove the *BsmBI* restriction site. This substitution was validated by Sanger sequencing and did not interfere with virus recovery. Hence, the modification was genetically stable and fully compatible with efficient virus replication. Although other studies have employed a GGA-based reverse genetics system to reconstruct a full-length IBV genome, this is the first to apply this strategy to a vaccine strain of IBV (H120). We acknowledge that whole-genome sequencing (WGS) would represent the most comprehensive method to exclude rare unintended mutations. However, successful rescue of infectious virus following transfection and serial passage in embryonated SPF chicken eggs, Detection of subgenomic mRNAs (genes 5 and 7) confirms correct transcription regulatory sequence (TRS) functionality and proper genome organization and Comparable replication kinetics, EID_50_ titers, and in vivo biological behaviour between rH120 and the parental H120 strain provide strong functional evidence that no biologically relevant defects occurred elsewhere in the genome.

The rescued rH120 virus replicated effectively in embryonated SPF chicken eggs, reaching titers comparable to those of the parental commercial H120 strain. The mean titer of approximately 10^5^ EID_50_/mL reflects robust viral propagation, suggesting that the recombinant virus retained the replication competence of the original strain. This concurs with earlier studies on IBV, showing that carefully designed genome manipulation does not necessarily compromise viral growth characteristics [23,27,28].

Both the rH120 virus and the original H120 strain showed nearly identical replication kinetics and progressive increases in viral RNA up to 36 h post-infection followed by stabilization. The slightly higher RNA copy number observed for rH120 at 24 h (*p* < 0.05) may indicate a modest replication advantage during the early phase of infection, though this difference was transient [27]. Beyond 24 h, both viruses displayed equivalent growth patterns, confirming that the introduced modification did not affect the overall replication profile. IBV infection is associated with early local inflammatory and cellular recruitment responses in the respiratory tract. A controlled level of early rH120 replication may provide sufficient innate stimulation for effective priming without excessive immunopathology, aligning with the design intent of attenuated live vaccines [29]. The biological stability of the recombinant construct and its suitability for further functional and immunological evaluations have been reported in studies of related coronaviruses such as SARS-CoV-2 and Porcine Epidemic Diarrhoea Virus [20,30].

The broiler chickens produced no deleterious clinical indications following experimental infection, indicating the in vivo safety and tolerance of rH120 and H120 [25,31]. The viral shedding profiles in throat and cloacal swabs followed a typical IBV infection pattern, peaking approximately 6 days post-infection and declining by day 10 [32]. The consistently higher RNA levels detected in throat swabs compared with cloacal samples reflect the respiratory tropism of IBV and align with previous reports indicating limited intestinal replication [27]. The comparable shedding dynamics between the two groups further demonstrate that rH120 preserves the in vivo replication characteristics the original H120 strain.

The virological results were consistent with the serological findings. Both rH120 and H120 induced strong antibody responses detectable by 7 days post-infection, with antibody levels continuing to rise by day 14 [14,31]. The slightly higher mean ELISA optical density values in the rH120 group indicate that the recombinant virus may stimulate a modestly stronger humoral immune response. This enhanced immunogenicity may reflect improved antigen expression or subtle differences in replication dynamics [33]. Additional challenge studies will be necessary to determine whether this response translates into increased protective efficacy. Consistent with published reverse-genetics recombinant H120 vaccines, IBV-specific ELISA responses typically become detectable or rise markedly between 7 and 14 days post-vaccination, and challenge performed at 14 dpv frequently demonstrates measurable protection (e.g., survival/clinical protection and reduced lesions and/or shedding) [34,35]. Serological monitoring by ELISA does not, on its own, establish protective immunity; however, it is an accepted and widely applied indicator of vaccination response (vaccine take) and the magnitude/uniformity of humoral immunogenicity following IBV vaccination, including recombinant IBV vaccine constructs [33,36].

Overall, the reconstruction and rescue of rH120 represent a major advance in the development of a flexible reverse genetics platform for IBV. The recombinant virus demonstrated genetic stability, biological competence, and immunogenicity in vivo. These findings provide a foundation for future work including rational attenuation, chimeric vaccine development. and investigations into spike gene function and viral pathogenesis. Future studies will incorporate homologous and heterologous challenge experiments, complementary assays for infectious virus quantification, and evaluation of cellular immune responses to further define the protective profile of the rH120 vaccine and will explore the use of this platform to engineer next-generation IBV vaccines with improved cross-protective capacity.

## 5. Conclusions

This study reports the successful construction and rescue of a recombinant Infectious Bronchitis Virus (rH120) using a Golden Gate Assembly reverse genetics system. The recombinant virus exhibited genetic stability, replicated efficiently in embryonated SPF chicken eggs, and displayed biological characteristics comparable to the original H120 strain. In vivo, rH120 was safe, showed predominant replication in the respiratory tract, and elicited a strong antibody response in broiler chickens. The Golden Gate Assembly-based platform provides a rapid, scalable, and cost-effective strategy for generating genotype-matched IBV vaccines, enabling precise genetic modification and rapid updating of vaccine strains in response to emerging variants. The approach is directly compatible with industrial vaccine development pipelines and supports the development of next-generation live-attenuated and recombinant IBV vaccines with enhanced cross-protective potential.

## Data Availability

The original contributions presented in this study are included in the article. Further inquiries can be directed to the corresponding author.

## Abbreviations

The following abbreviations are used in this manuscript:

IBV	Infectious Bronchitis Virus
rH120	recombinant H120
SPF	Specific-Pathogen-Free
GGA	Golden Gate Assembly
CEFs	chicken fibroblast cells
UTR	Untranslated region
HDZ	Hepatitis Delta Virus
cDNA	Complementary DNA
NFW	Nuclease-free water
dpi	Days post-infection
dpv	Days post-vaccination
EID50.	Embryo Infectious Dose 50
RT-qPCR	Reverse Transcriptase-Quantitative PCR
